# Association between G6PD deficiency and schizophrenia A case report

**DOI:** 10.1192/j.eurpsy.2024.1013

**Published:** 2024-08-27

**Authors:** B. Abassi, F. Fekih-Romdhane, F. Baccar, M. Cheour, R. Damak, S. Ellini

**Affiliations:** ^1^Ibn Omrane, Razi Hospital, Mannouba, Tunisia

## Abstract

**Introduction:**

G6PD is essential for the production of NADPH, which is a cofactor for many enzymes involved in antioxidant defense and neurotransmitter synthesis. A deficiency in this enzyme could lead to increased oxidative stress, impaired neurotransmitter and immune function. The latter have been implicated in the pathophysiology of schizophrenia.

**Objectives:**

The present case is presented to underscore the infrequent and uncharacteristic manifestation of this condition, in the context of clinical symptoms and the trajectory of evolution of schizophrenia when associated with G6PD Deficiency. Moreover, it sheds light on the challenges clinicians encounter in the management of such cases.

**Methods:**

A case report of a patient who was admitted to the Psychiatry Department (“Ibn Omrane”) of Razi Hospital”.

**Results:**

Mr. M.T is a 26 year-old unmarried man. He comes from a non-consanguineous marriage and has an educational level of a bachelor’s degree plus three additional years of study. He has a significant family medical history. His maternal uncle is under treatment for a chronic psychotic disorder. He has a personal history of G6PD deficiency and no specific habits to note. At the age of 24, he insidiously developed anxiety with incoherent statements of persecution accompanied by behavioral manifestations leading to mistrust and social isolation. He discontinued his studies for a year and began verbalizing suicidal thoughts accompanied by self-harm behaviors.

The family sought help from a psychiatrist who prescribed 5 mg of olanzapine, which was covertly administered to the patient.

At the age of 28, after a suicide attempt, he was involuntarily admitted to Razi Hospital. The clinical presentation was dominated by disorganization, with a partial response to treatment.

**Conclusions:**

More research is needed to confirm the association between G6PD deficiency and schizophrenia and to determine the underlying mechanisms. Larger studies with well-defined populations and methodologies are needed. It is also important to study the interaction between G6PD deficiency and other genetic and environmental factors that contribute to schizophrenia.

**Disclosure of Interest:**

None Declared

